# Oral submucous fibrosis: a contemporary narrative review with a proposed inter-professional approach for an early diagnosis and clinical management

**DOI:** 10.1186/s40463-020-0399-7

**Published:** 2020-01-08

**Authors:** Naman R. Rao, Alessandro Villa, Chandramani B. More, Ruwan D. Jayasinghe, Alexander Ross Kerr, Newell W. Johnson

**Affiliations:** 1 Harvard Medical School, Harvard University, Boston, MA USA; 2 Division of Oral Medicine and Dentistry, Brigham and Women’s Hospital, Boston, MA USA; 3 Harvard School of Dental Medicine, Boston, MA USA; 4 Department of Oral Medicine and Radiology, K. M. Shah Dental College and Hospital, Sumandeep Vidyapeeth University, Vadodara, Gujarat India; 5 Department of Oral Medicine and Periodontology, Faculty of Dental Sciences, University of Peradeniya, Peradeniya, Sri Lanka; 6 Department of Oral and Maxillofacial Pathology, Radiology and Medicine, NYU College of Dentistry, New York, NY USA; 7 Menzies Health Institute Queensland, Griffith University, Southport, Queensland Australia

**Keywords:** Oral submucous fibrosis, Global epidemiology, Areca nut, Management

## Abstract

Oral Submucous fibrosis (OSMF) has traditionally been described as “a chronic, insidious, scarring disease of the oral cavity, often with involvement of the pharynx and the upper esophagus”. Millions of individuals are affected, especially in South and South East Asian countries. The main risk factor is areca nut chewing. Due to its high morbidity and high malignant transformation rate, constant efforts have been made to develop effective management. Despite this, there have been no significant improvements in prognosis for decades. This expert opinion paper updates the literature and provides a critique of diagnostic and therapeutic pitfalls common in developing countries and of deficiencies in management. An inter-professional model is proposed to avoid these pitfalls and to reduce these deficiencies.

## Introduction

Oral Submucous Fibrosis (OSMF) is a potentially malignant disorder which was described by Schwartz in 1952 as “*Atropica idiopathica mucosae oris*” and later by Jens J. Pindborg in 1966 as “an insidious, chronic disease that affects any part of the oral cavity and sometimes the pharynx [1]. Although occasionally preceded by, or associated with, the formation of vesicles, it is always associated with a juxtaepithelial inflammatory reaction followed by fibroelastic change of the lamina propria and epithelial atrophy that leads to stiffness of the oral mucosa and causes trismus and an inability to eat” [1]. OSMF is also characterized by reduced movement and depapillation of the tongue, blanching and leathery texture of the oral mucosa, progressive reduction of mouth opening, and shrunken uvula [2–4]. Other terms used to describe OSMF include idiopathic scleroderma of the mouth, juxtaepithelial fibrosis, idiopathic palatal fibrosis, diffuse oral submucous fibrosis, and sclerosing stomatitis [5–8].

### Epidemiology (Table 1) (Fig. 1)

Worldwide, the number of cases of OSMF was estimated to be 2_._5 million in 1996 [33]. Although many case finding studies have been conducted, particularly in South and South East Asia, OSMF is not a notifiable disease and no population-based data are available [33]. The prevalence of OSMF in India has been estimated to range from 0.2–2.3% in males and 1.2–4.6% in females, with a broad age range from 11 to 60 years [34–36]. A marked increase in incidence has been observed after the widespread marketing of commercial tobacco and areca nut products, generally known as Gutkha, which is sold in single-use packets [33]. Currently, it is estimated that areca nut is consumed by 10–20% of the World’s population in a wide variety of formulations [37, 38]. The global South Asian diaspora also has a significant problem with cases reported from the United Kingdom, USA, South Africa, and many European countries.

**Table 1:** Worldwide prevalence studies on Oral Submucous Fibrosis

Year	Authors	Study type	Sample size	Country	City/district	State/Province	Prevalence (%)
1965	Pindborg J. J. et al. [9]	Observational	10,000	India	Mumbai	Maharashtra	0.50
1965	Pindborg J. J. et al. [10]	Cross sectional	10,000	India	Lucknow	Uttar Pradesh	4.1
1966	Pindborg J. J. et al. [11]	Observational	10,000	India	Bengaluru	Karnataka	0.18
1966	Zachariah et al. [12]	Observational	5000	India	Thiruvananthapuram	Kerala	1.22
1968	Pindborg J. J. et al. [13]	Observational	50,915	India	Srikakulam	Andra Pradesh	0.04
					Darbhanga	Bihar	0.07
					Bhavnagar	Gujarat	0.16
					Ernakulum	Kerala	0.36
1970	Wahi et al. [14]	Observational		India	Mainpuri	Uttar Pradesh	0.59
1972	Mehta F. S. et al. [15]	Survey	101,761	India	Pune	Maharashtra	0.03
1982	Lay K. M. et al. [16]	Cross sectional	6000	Myanmar	Bilugyun	Mon	0.1
1988	Seedat H. A. et al. [17]	Cross sectional	2400	South Africa	Durban	KwaZulu-Natal	3.4
1997	Tang J.G. et al. [18]	Cross sectional	11,046	China	Xiangtan	Hunan	3.30
2006	Patil P. B. et al. [19]	Cross sectional	2400	India	Dharwad	Karnataka	7.8
2007	Hazarey V. K. et al. [20]	Cross sectional	1000	India	Nagpur	Maharashtra	6.42
2008	Mathew A. L. et al. [21]	Observational	1190	India	Manipal	Karnataka	2.01
2008	Mehrotra R. et al. [22]	Retrospective	1151	India	Allahabad	Uttar Pradesh	17.02
2012	Sharma R. et al. [23]	Cross sectional survey	6800	India	Jaipur	Rajasthan	3.39
2012	Agarwal A. et al. [24]	Observational	750	India	Dehradun	Uttarakhand	5.4
2013	Bhatnagar P. et al. [25]	Survey	8866	India	Modinagar	Uttar Pradesh	1.97
2014	Burungale S. U. et al. [26]	Cross sectional	800	India	Jaitala, Nagpur	Maharashtra	2.62
2014	Nigam N. K. et al. [27]	Observational	1000	India	Moradabad	Uttar Pradesh	6.3
2015	Patil S. et al. [28]	Observational	5100	India	Jodhpur	Rajasthan	30
2016	Singh P. et al. [29]	Cross sectional survey	132	India	Nagpur	Maharashtra	2.86
2018	Tyagi V. N. et al. [30]	Cross sectional	1167	India	Nashik	Maharashtra	3.51
2018	Yang S. F. et al. [31]	Cross sectional	23,373,51	Republic of China	–	Taiwan	16.2
2019	More C. B, et al. [32]	Cross sectional	13,874	India	Vadodara	Gujarat	7.21

**Fig. 1:**
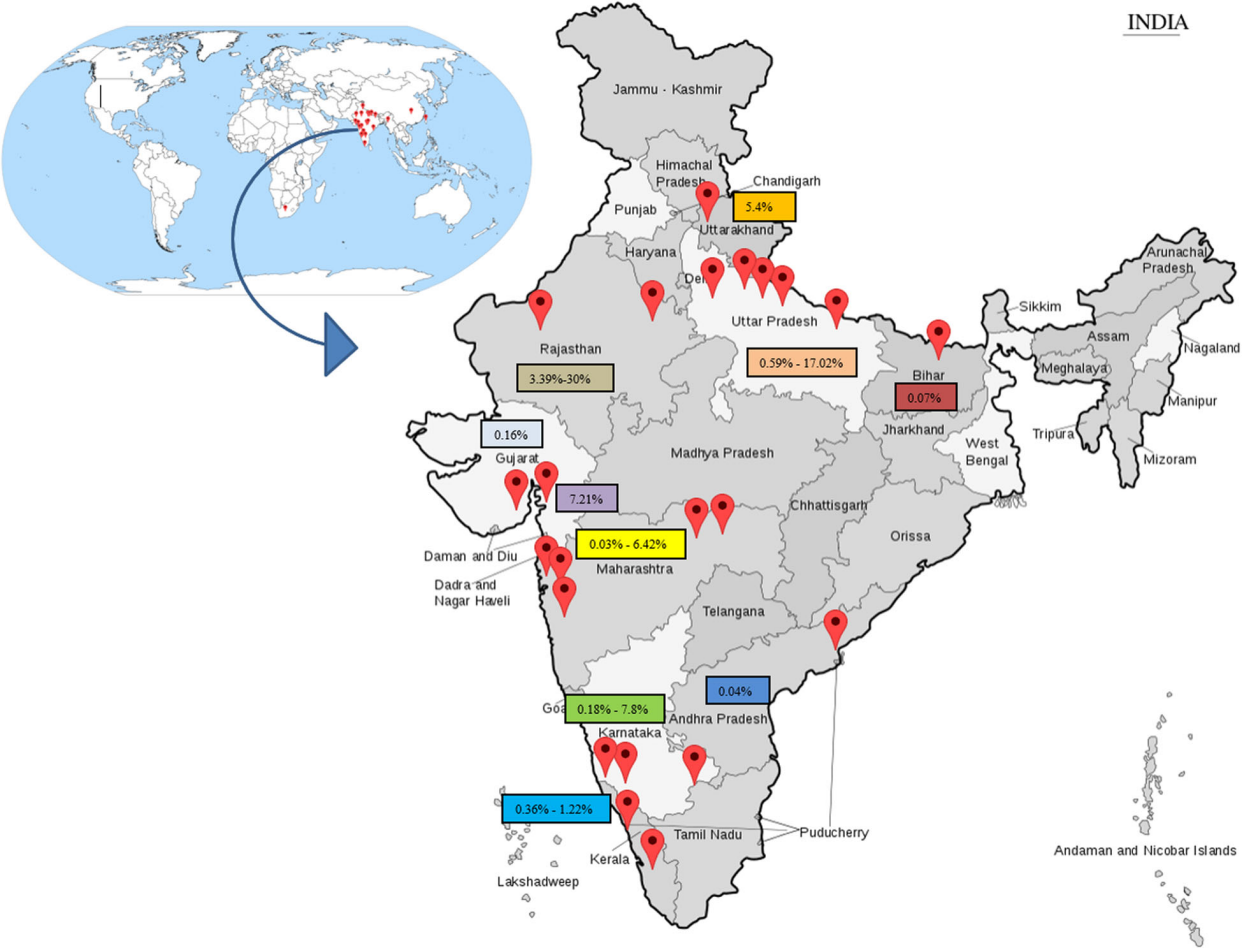
Global and Indian prevalence studies of Oral Submucous Fibrosis

Table 1 and Fig. 1 present published estimates of the prevalence of OSMF, which range from 0.1 to 30%, varying by geographical location, sample size, and sampling methodology. There is an urgent need for large well-designed epidemiological surveys to understand the true global and regional burden of OSMF.

### Major etiology, contributing factors and etiopathogenesis (Tables 2 and 3) (Fig. 2)

**Fig. 2:**
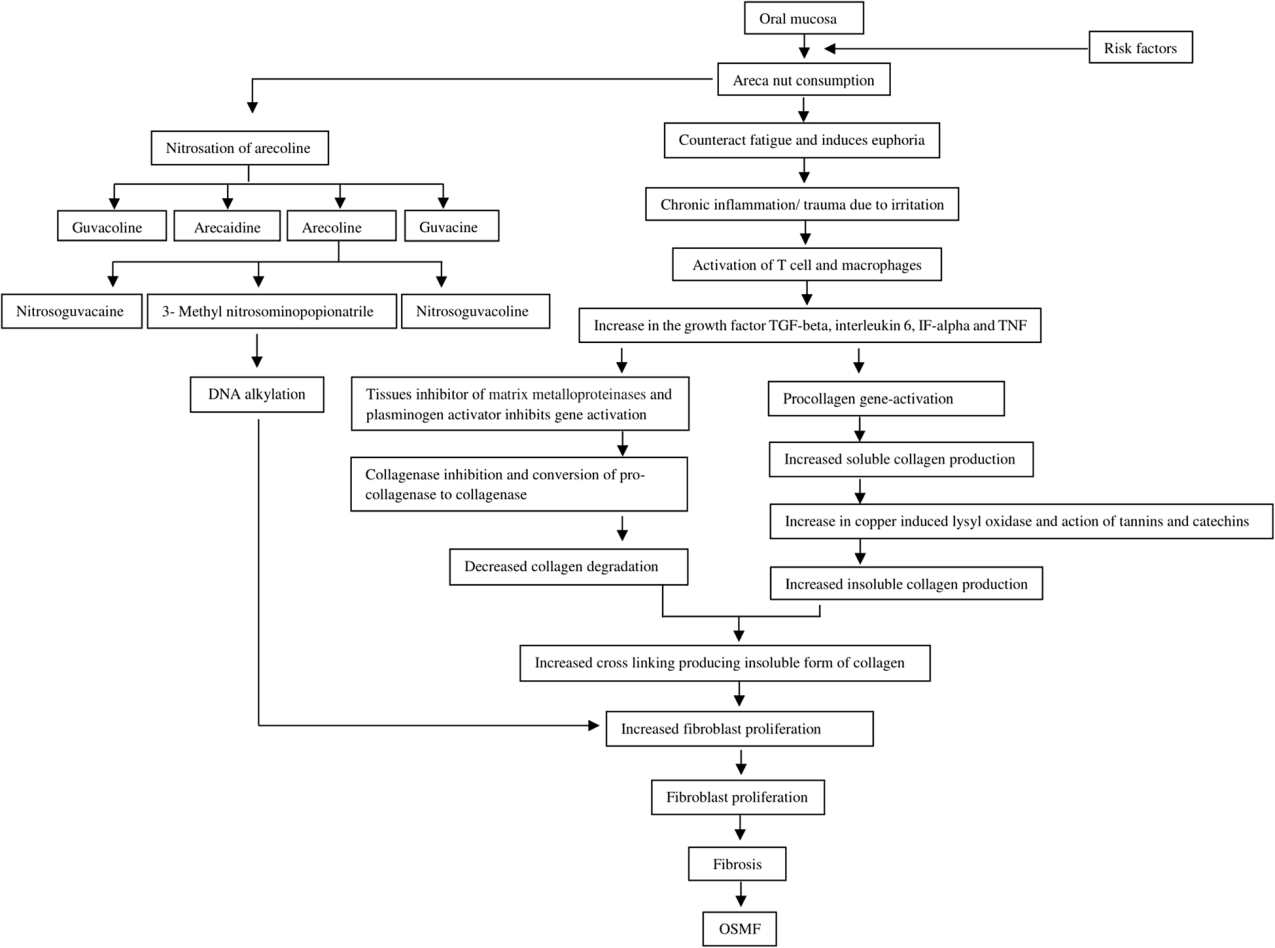
Etiopathogenesis [44]

Although the etiopathogenesis of this disease is multifactorial, areca nut-chewing in any formulation is considered the main causative agent. (Fig. 2) Contributory risk factors suggested includes chewing of smokeless tobacco, high intake of chilies, toxic levels of copper in foodstuffs and masticatories, vitamin deficiencies, and malnutrition resulting in low levels of serum proteins, anemia and genetic predisposition.

**Table 2:** Major aetiology of Oral Submucous Fibrosis

Major aetiology	Description
Chewing of Areca nut (Baked or Raw) and/or derivatives such as Gutkha, Pan masala, Mawa, Betel quid, Sweet Supari and other formulations.	Arecoline and Arecaidine nitrosation causes DNA alkylation with proliferation of fibroblasts and elevated collagen synthesis [39].

**Table 3:** Contributing risk factors for Oral Submucous Fibrosis

Contributing factors	Description
Chewing smokeless tobacco	Dip, Snuff, Snus and chewing tobacco have been reported as major contributing factors [34, 35, 39, 40].
Nutritional	Deficiencies of iron, folate & vitamin B12 result in mucosal atrophy, notably in the mouth. Increased levels of iron enhance hydroxylation of proline and lysine in the process of collagen synthesis [40].
Chilies	Hypersensitivity reactions to capsaicin might contribute to fibrosis [41–43].
Toxic levels of copper	Copper upregulates the enzyme lysyl oxidase, enhancing cross linking of collagen and elastin [35, 44, 45].
Genetic predisposition	HLA-A10, HLA-B7, HLA-DR3, haplotypes A10/DR3, B3/DR3 and A10/B8 are found in increased frequency in OSMF patients [45].
Immunological predisposition	Subjects with high endogenous expression of CD4 and HLA-DR on lymphocytes and Langerhans cells may have dysregulation of their immune-inflammatory response with bystander tissue injury [45].

### Diagnostic approach

**Fig. 3:**
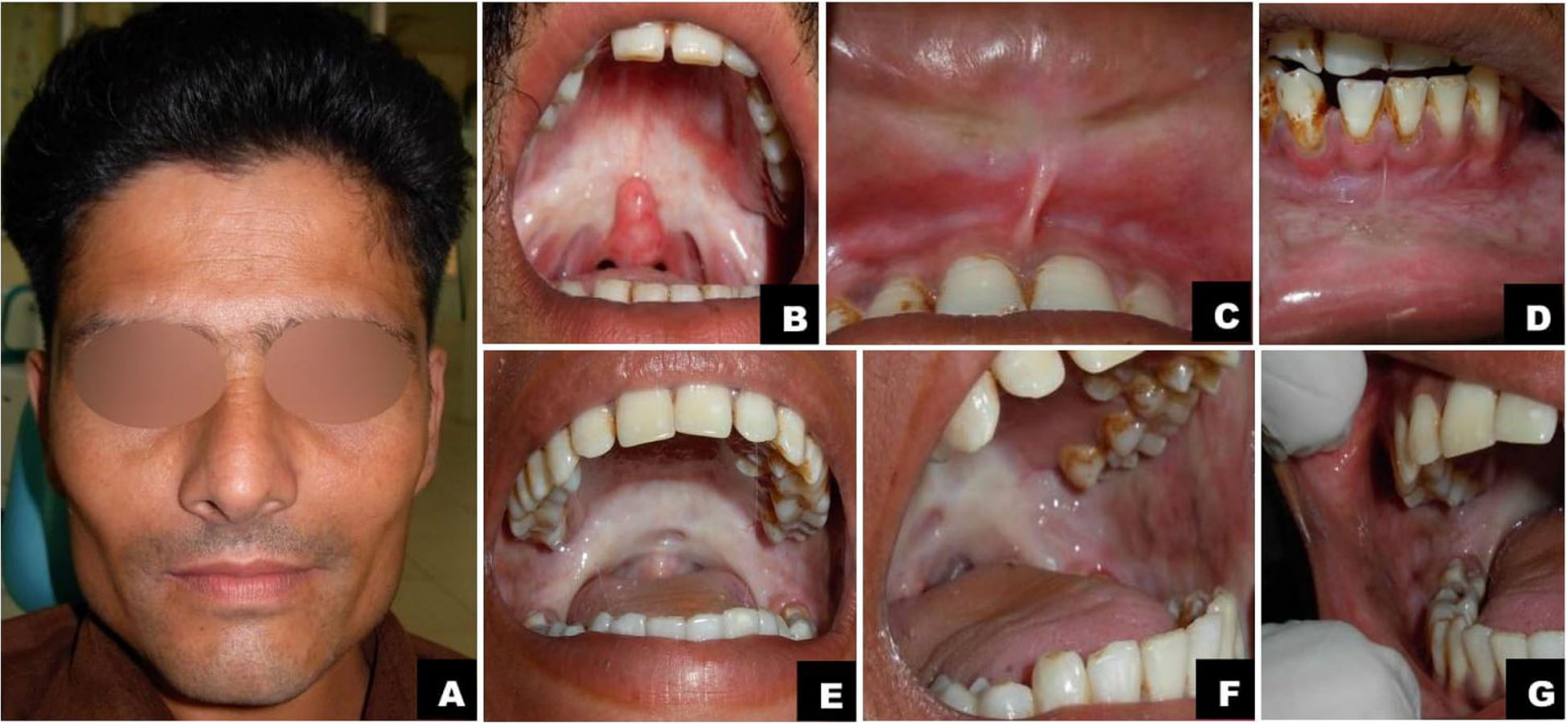
Clinical expressions of Oral Submucous Fibrosis. Oral Submucous Fibrosis in a 27-year-old male with a history of gutkha chewing. Panel A shows sunken cheeks and prominent malar bone. Panel B shows significant blanching or marble-like appearance of the soft palate and faucial pillars. Note the altered, inverted shape of the uvula. Panels C & D show blanched bands of upper and lower labial mucosae and vestibule, which are stiff and palpable. Panels E, F & G: A 24-year-old female with a history of chewing baked areca nut. Panel E: significant blanching of soft palate and faucial pillars, and shrunken uvula. Panels F & G: thick fibrous bands and brown/black pigmentation on left & right buccal mucosae

Diagnosis of OSMF is based on clinical signs and symptoms that include burning sensation, pain, and ulceration (Table 4) [4, 46, 47]. Progressive restriction in mouth opening, blanching of the mucosa, depapillation of the tongue, and loss of pigmentation are other classic features (Fig. 3) [46]. Dysphonia and hearing impairment is also observed in advanced cases [48, 49]. Quality of life (QoL) is severely affected, worsening with increasing stage of the disease [50].

**Table 4:** Intra- and extra- oral manifestations of OSMF at different stages

Features	Early stage	Moderate stage	Advanced stage
Intra oral	Stomatitis, excessive salivation, burning sensation, blanching of oral mucosa, blister formation, presence of thin palpable fibrous bands, sparse brown/black pigmentation.	Stomatitis, burning sensation, xerostomia, loss of taste sensation, gradual decrease in mouth opening, difficulty in whistling, vesicle formation, petechiae, rigid oral mucosa, difficulty in blowing the cheeks, defective gustatory sensation, blanching of oral mucosa – especially of soft palate, buccal mucosa, labial mucosa, tongue, floor of mouth, and faucial pillars. Presence of thick palpable fibrous bands, shrunken uvula with altered shape (inverted, hockey stick, bud like, deviated).	Stomatitis, burning sensation, xerostomia, reduction in mouth opening, restricted tongue movement, loss of taste sensation, Unable to blow the cheeks, defective gustatory sensation, inability to whistle, blanching of oral mucosa: esp. soft palate, buccal mucosa, labial mucosa, tongue, floor of mouth, and faucial pillars. Loss of suppleness of mucosa, mottled or opaque or white marble like appearance of oral mucosa, thick palpable fibrous bands on buccal and labial mucosa, de-papillation of tongue, shrunken uvula with altered shape (inverted, hockey stick, bud like, deviated), involvement of the pharyngeal and esophageal mucosa.
Extra oral	No Significant extra oral features are observed.	Prominent masseter muscle, nasal twang, sunken cheeks, thinning of lips, difficulty in deglutition, loss of naso-labial fold, prominent antegonial notch, hoarseness of voice, mild hearing impairment, weight loss.	Hypertrophic and stiff masseter muscle, nasal intonation of voice, sunken cheeks, multiple folds on cheeks when attempting wide opening of mouth, thinning of lips, difficulty in deglutition, loss of naso-labial fold, prominent antegonial notch, hoarseness of voice, severe hearing impairment, severe weight loss, hoarseness of voice, difficulty in deglutition, atrophy of facial musculature. In severe cases, radiographically, there is alteration in condylar form and fibrous ankylosis of the temporomandibular joints.

OSMF progresses over time and management depends on the stage at clinical presentation. In 2012, More et al. proposed a disease progression-based classification (Table 5) which represents the clinical and functional staging of OSMF. This classification has been widely accepted/recommended as the closest fit for Indian population, especially to understand the disease progression/ clinical pattern [3, 35, 51]. In 2017, Passi D. et al. proposed a pathologically updated and treatment management-based classification. This classification chiefly focuses and recommends the treatment management based on the clinical stage of OSMF [52]. Later in 2018, Arakeri G. et al. proposed a three-component classification scheme (TFM) which can essentially be useful for effective communication amongst the care team, categorization of OSMF, recording data and disease prognosis, and treatment management. Additionally, this classification also describes OSMF malignant transformation in detail [53].

**Table 5:** More et al. 2012 classification of OSMF

Clinical staging	Interpretation
Stage 1 (S1)	Stomatitis and/or blanching of oral mucosa.
Stage 2 (S2)	Presence of palpable fibrous bands in buccal mucosa and/or oropharynx, with /without stomatitis.
Stage 3 (S3)	Presence of palpable fibrous bands in buccal mucosa and/or oropharynx, and in any other parts of oral cavity, with/ without stomatitis.
Stage 4 (S4)	Any one of the above stages along with other potentially malignant disorders (e.g. oral leukoplakia, oral erythroplakia)
	Any one of the above stages along with oral squamous cell carcinoma.
Functional staging	Interpretation
M1 Staging	Interincisal mouth opening up to or greater than 35 mm.
M2 Staging	Interincisal mouth opening between 25 and 35 mm.
M3 Staging	Interincisal mouth opening between 15 and 25 mm.
M4 Staging	Interincisal mouth opening less than 15 mm.

### Approaches to non-surgical management.

Although there is general agreement regarding clinical staging, approaches to management of patients vary widely [54]. Numerous interventions have been reported and are summarized in Table 6 [60, 68–70]. Supportive regimens, such as vitamin and iron supplements, a mineral-rich diet, red fruits, green leafy vegetables, and green tea consumption, are often recommended but there are no good quality studies confirming their efficacy.

**Table 6:** Treatments for OSMF

Treatment type	Agent	Authors	Study Type	Sample size (n)	Main findings
Antioxidant treatments	Lycopene	Karemore T. V. and Motwani M [55].	Single blinded prospective study	92	Ingestion of 8 g/QD of lycopene (*n* = 46) for three months was shown to be effective in the reduction of burning mouth and mouth opening (*p* < 0.05) in patients with OSMF when compared to the placebo group (*n* = 46).
	Curcumin	Hazarey V. et al. [56]	Randomized control clinical trial	30	Sucking 2 g/QD of Curcumin lozenges (*n* = 15) with physiotherapy for three months showed a significant improvement in both mouth opening and in alleviating the burning sensation (*p* < 0.05) in comparison to the control group (clobetasol propionate 0.05%; (*n* = 15).
	Micronutrient therapy	Maher R. et al. [57]	Single arm preliminary prospective study	117	Swallowing micronutrient supplements: vitamins A, B complex, C, D, E; and minerals iron, calcium, copper, zinc, and magnesium was observed to be significantly effective (p < 0.05) in reduction of sign and symptoms of OSMF over 3 years.
	Spirulina and *Aloe Vera*	Patil S. et al. [58]	Double blinded prospective study	42	Ingestion of 500 mg/QD of Spirulina (*n* = 21) for 3 months was associated with a significant improvement in mouth opening and reduction in ulcers/erosions/vesicles (*p* < 0.05) in comparison to 5 mg of aloe vera (*n* = 21) for the same time. Improvement in burning sensation and pain associated with lesions was not found significant between two groups
		Alam S. et al. [59]	Double-blinded, placebo- controlled, parallel-group randomized controlled trial	60	Application of aloe vera gel over buccal mucosa, palate, retromolar region, and floor of the mouth twice daily during submucosal injection of hyaluronidase and dexamethasone (*n* = 15) and surgical treatment (buccal fat pad, nasolabial flap, or collagen membrane, (*n* = 15) treatment with 6 months of follow up was observed to be a significant adjuvant therapy in reduction of most of the symptoms of OSMF (*p* < 0.01), in comparison to a similar group of medicines alone, (*n* = 15) and surgical procedures (*n* = 15)] with no application of aloe vera.
Medicinal treatments	Steroids	Goel S. et al. [60]	Longitudinal prospective study	270	4 mg/ml/biweekly injections of Betamethasone diluted in 1.0 ml of 2% xylocaine for 6 months given on buccal mucosa, bilaterally, using an insulin syringe, with a half dose on each side, was showed significant improvement of mouth opening and reduction in burning sensation in a stage II and stage III OSMF group (*p* < 0.0001), in comparison to a control group which received no treatment over two years.
	Hyaluronidase	James L. et al. [61]	Retrospective study	28	Intralesional injection of Hyaluronidase 1500 IU mixed in 1.5 ml of dexamethasone and 0.5 ml of lignocaine hydrochloride biweekly for 4 weeks showed a significant improvement in mouth opening with net gain of 6 ± 2 mm (92%), reducing the burning sensation (89%), number of painful ulceration (78%) and blanching of oral mucosa (71%) for Grade III OSMF patients.
	Colchicine + Hyaluronidase	Krishnamoorthy B. & Khan M [62].	Comparative prospective study	50	1 mg/ day colchicine tablet and 0.5 ml intralesional Injection hyaluronidase 1500 IU/ once a week (group I, *n* = 25) for twelve weeks showed a significant improvement in mouth opening (*p* < 0.05) and reduced burning sensation (33% by second week) in comparison to subjects treated with 0.5 ml intralesional injection of hyaluronidase 1500 IU and 0.5 ml intralesional injection hydrocortisone acetate 25 mg/ml once a week alternatively (group II, *n* = 25).
	Placental extracts	Singh P. et al. [63]	Comparative prospective study	10	2 ml intralesional placental extract mixed with 2 ml of 2% lignocaine HCL weekly for an interval of 8 weeks showed an average improvement in mouth opening by 8.02 mm (average pretreatment mouth opening = 18.49 mm, average posttreatment mouth opening = 26.51 mm) with average marked reduction in burning sensation by 4.9 (average pretreatment burning sensation = 8.0, average posttreatment burning sensation = 3.1). Burning sensation was assessed using visual analogue scale with 0–10, where 0 = no burning sensation and 10 = maximum burning sensation.
	Isoxupurine	Bhadage C. J. et al. [64]	Prospective study	40	10 mg Isoxsuprine tablets/ QID with oral physiotherapy (Group A, *n* = 15) plus 2 ml dexamethasone by intralesional injection with 1500 IU hyaluronidase mixed with 1 ml of 2% lignocaine solution with adrenaline 1:80,000 (Group B, n = 15) for six weeks with a follow up of 4 months, showed a significant improvement in mouth opening (*p* < 0.05) and burning sensation (*p* < 0.00001) in comparison to the placebo group (only oral physiotherapy) (Group C, *n* = 10).
	Pentoxifylline	Rajendran R. et al. [65]	Randomized controlled clinical trial	29	400 mg/ TID of Pentoxifylline tablets (*n* = 14) for seven months showed a significant improvement in mouth opening (*p* < 0.0001), tongue protrusion (*p* < 0.05), relief from perioral fibrotic bands (*p* < 0.0001), subjective symptoms of intolerance to spices (*p* < 0.0001), burning sensation of mouth (p < 0.0001), tinnitus (*p* < 0.0001), difficulty in swallowing (*p* < 0.0001) and difficulty in speech (*p* < 0.0001) in comparison to the control group (multivitamin with local heat therapy, *n* = 15).
Oral physiotherapy	Ultrasound + Physiotherapy	Kumar V. et al. [66]	Single arm prospective study	15	Ultrasound therapy with 0.7–1.5 W/Cm^2^ with thumb kneading physiotherapy for six days/ week for two consecutive weeks showed significant improvement in mouth opening (*p* < 0.001) and reduction of burning sensation.
Surgical approaches	Surgery	Kamath V. V [67].	Systematic Review	–	Lasers, tongue flap, palatal flap, buccal fat pad, nasolabial flap, thigh flaps, split skin grafts, collagen membrane, artificial dermis, human placenta grafts, coronoidectomies, muscle myotomies and oral stents. All surgeries have shown significant improvement in the symptoms of OSMF. However there exist no definite protocols and thus author comments that treatment remains subjective to the operating surgeon.

### Malignant transformation of OSMF

OSMF is classified as an oral potentially malignant disorder (OPMD) [3]. Patients with OSMF have been reported with higher risk of developing oral squamous cell carcinoma (OSCC), compared to other OPMD’s [71, 72]. Although 7.6% of OSMF cases transformed to oral squamous cell carcinoma (OSCC) in a 17-year follow up study reported in 1970 [73], other studies with smaller follow up periods report malignant transformation rates ranging from 1.9–9%, [74–76] depending on diagnostic criteria and duration of follow up [77].

Studies suggest that malignant transformation in patients with OSMF differs from those without OSMF. This difference is believed to arise from the mechanism of areca nut carcinogenesis. A retrospective study conducted in China reported that oral cancer originating from OSMF is clinically more invasive and exhibits higher metastasis and recurrence rates compared to “conventional” OSCC [78]. In contrast, Chaturvedi et al. found that OC arising in a background of OSMF represented a clinico-pathologically distinct entity, less aggressive than the “conventional” tobacco-related OC’s seen in India [46]. Better prognostic features associated with OC occurring in a background of OSMF included early tumor stage, thinner lesions, fewer neck metastases with less extra-capsular spread, and more highly differentiated neoplasms. It was suggested that fibrosis in the oral mucosa and tumor stroma, with reduced vascularity, inhibits lymphatic and vascular spread [46].

Studies have shown higher risk of malignant transformation of OSMF when observed with simultaneous oral leukoplakia [77]. A wide array of studies was implemented recently to determine the possible mechanisms involved in malignant transformation, and many have focused their attention on molecular markers which could be helpful for early diagnosis and have possible, helpful therapeutic implications [79–81].

### Proposed diagnostic and management approach

As with other lifestyle related diseases, primary prevention at population and individual levels needs to be improved. Space does not permit an exhaustive discussion of the approaches here but, in the case of OSMF, this involves education of the public regarding the dangers of areca nut and tobacco, and legislation to restrict the sale of gutkha and similar products [82–84]. Several Indian states have had success in this regard. Since May 2013, gutkha is banned in 24 states and 5 union territories of India, under the provision of centrally enacted Food Safety and Regulation (Prohibition) Act 2011 [85]. The ban is enforced by the State public health ministry, Food and Drug Administration and the local police. Although there is a significant reduction in the legal purchase of gutkha, the Supreme Court and higher enforcement bodies are still chasing to cease the illegal sale [85, 86].

**Fig. 4:**
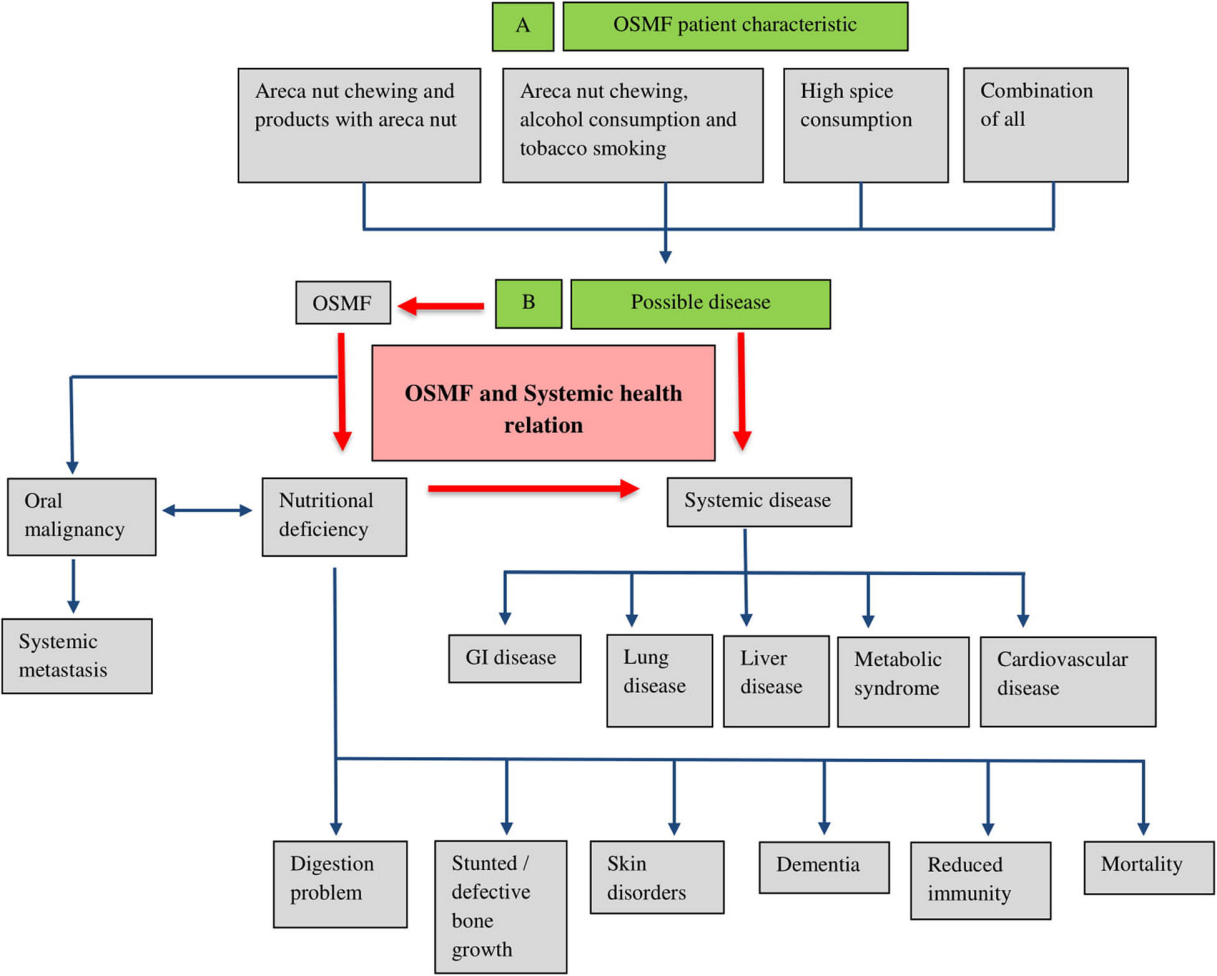
Oral and Systemic outcomes of OSMF possible in the absence of holistic management

What of the many millions already afflicted? Despite efforts to improve the management of OSMF, many come so late to diagnosis that cure is impossible, and interventions are of limited efficacy. So early diagnosis is of great importance. Further, most OSMF patients chew tobacco as well as an areca nut product, may imbibe unhealthy amounts of alcohol, and abuse other drugs. They often have dietary deficiencies. Therefore, they are at high risk of co-morbidities, including metabolic syndromes, respiratory, gastrointestinal/liver and cardiovascular diseases. (Fig. 4) [87, 88].

Dependent on their dominant symptoms, patients may seek consultation from either primary care physicians (PCP) or dentists. When examined by a dentist, the diagnostic and treatment approach is likely to be focused on the oral signs and symptoms. Conversely, when patients present to a PCP, the focus of management is likely to be general, with the oral condition under-investigated and under-managed. In most of the world, these patients are not managed by a multidisciplinary team.

We propose an inter-professional approach that may increase rates of early diagnosis of OSMF and potentially malignant disorders/OSCC, with integrated management of both oral and systemic symptoms, improving long-term prognosis, reducing suffering and improving quality of life.

When a patient presents to a dentist, and a clinical diagnosis of OSMF is made, he/she should be referred to their primary care physician with a note of planned dental management. If any underlying systemic disease is diagnosed, the medical treatment plan should be communicated back to the dentist. If no systemic disease is diagnosed, a written medical clearance letter, including an assessment of risks of developing any systemic condition, and recommendations for review visits, should be included.

When a patient presents to a physician, if he/she is a user of areca nut, and especially if restricted mouth opening is present, he/she should be immediately referred to a dentist detailing any planned management of other disease. The dentist should report back to the physician with a treatment plan for OSMF, if present, or dental clearance letter with a suggested risk of developing OSMF or any other oral disease.

This, after all, should be routine in any integrated health care system.

## Conclusion

Although studied intensively over many decades, one might say centuries, especially in South Asia, OSMF is hardly recognized and is poorly understood across the globe. The incidence is rising; there has been no significant improvement in management, nor reduction in its high malignant transformation rate.

Better integration of medical and dental services, especially in developing countries, may reduce patients’ suffering and improve their life quality. All health care professions must work together in public education and primary prevention.

## Data Availability

Not applicable.
